# Evolution of Dimethylsulfoniopropionate Metabolism in Marine Phytoplankton and Bacteria

**DOI:** 10.3389/fmicb.2017.00637

**Published:** 2017-04-19

**Authors:** Hannah A. Bullock, Haiwei Luo, William B. Whitman

**Affiliations:** ^1^Department of Microbiology, University of Georgia, AthensGA, USA; ^2^School of Life Sciences, The Chinese University of Hong KongHong Kong, Hong Kong

**Keywords:** DMSP, dimethylsulfoniopropionate, evolution, phytoplankton, Roseobacter

## Abstract

The elucidation of the pathways for dimethylsulfoniopropionate (DMSP) synthesis and metabolism and the ecological impact of DMSP have been studied for nearly 70 years. Much of this interest stems from the fact that DMSP metabolism produces the climatically active gas dimethyl sulfide (DMS), the primary natural source of sulfur to the atmosphere. DMSP plays many important roles for marine life, including use as an osmolyte, antioxidant, predator deterrent, and cryoprotectant for phytoplankton and as a reduced carbon and sulfur source for marine bacteria. DMSP is hypothesized to have become abundant in oceans approximately 250 million years ago with the diversification of the strong DMSP producers, the dinoflagellates. This event coincides with the first genome expansion of the Roseobacter clade, known DMSP degraders. Structural and mechanistic studies of the enzymes of the bacterial DMSP demethylation and cleavage pathways suggest that exposure to DMSP led to the recruitment of enzymes from preexisting metabolic pathways. In some cases, such as DmdA, DmdD, and DddP, these enzymes appear to have evolved to become more specific for DMSP metabolism. By contrast, many of the other enzymes, DmdB, DmdC, and the acrylate utilization hydratase AcuH, have maintained broad functionality and substrate specificities, allowing them to carry out a range of reactions within the cell. This review will cover the experimental evidence supporting the hypothesis that, as DMSP became more readily available in the marine environment, marine bacteria adapted enzymes already encoded in their genomes to utilize this new compound.

## Introduction

Dimethylsulfoniopropionate (DMSP) was first identified in 1948 and has since been found to be not only abundant in marine surface waters but also a valuable resource for many marine organisms and an integral part of the global sulfur cycle (Challenger and Simpson, 1948; van Duyl et al., 1998; Stefels et al., 2007). DMSP is the precursor of the climate-active gas dimethyl sulfide (DMS), which upon release into the atmosphere aids in the formation of cloud condensation nuclei (Lovelock et al., 1972; Hatakeyama et al., 1982). Additionally, DMS is the largest natural source of sulfur to the atmosphere, comparable in magnitude to the sulfur dioxide formed during the burning of coal. As DMS oxidation products display a longer residence time in the atmosphere than anthropogenic sulfur dioxide, their contribution to the global sulfur burden is also greater (Lovelock et al., 1972; Chin and Jacob, 1996).

**FIGURE 1:**
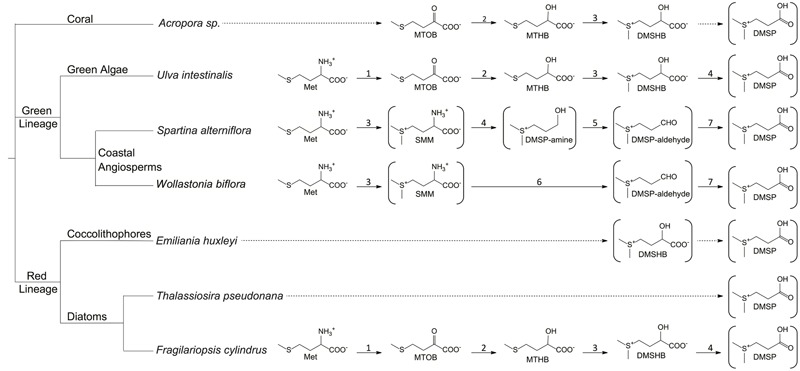
**Proposed DMSP biosynthetic pathways.** Structures in brackets have been detected and verified. Complete arrows signify reactions that are identified or predicted based on the observed intermediates. Dotted arrows signify unknown reactions. 1, aminotransferase; 2, NADPH-reductase; 3, methyltransferase; 4, decarboxylase; 5, oxidase; 6, decarboxylase/transaminase; 7, dehydrogenase. MTOB, 4-methylthio-2-oxobutyrate; MTHB, 4-methylthio-2-hydroxybutyrate; DMSHB, 4-dimethylsulfonio-2-hydroxybutyrate; SMM, *S*-methyl-L-methionine. (Hanson et al., 1994; James et al., 1995; Kocsis et al., 1998; Summers et al., 1998; Kocsis and Hanson, 2000; Lyon et al., 2011; Raina et al., 2013).

From an organismal viewpoint, DMSP is equally important. The ability to produce and metabolize DMSP is concentrated into specific classes of life. The main producers of DMSP are phytoplankton, mostly the classes Dinophyceae (dinoflagellates) and Prymnesiophycaea (coccolithophores) (Keller, 1989). DMSP production has also been noted in diatoms (Lyon et al., 2011; Kettles et al., 2014), the green algae *Ulva intestinalis* (Gage et al., 1997), corals (Raina et al., 2013), and certain higher plants like sugarcane (Paquet et al., 1994), and the coastal angiosperms *Spartina alterniflora* (Kocsis et al., 1998) and *Wollastonia biflora* (Hanson et al., 1994). Recently, DMSP biosynthesis was detected in several marine Alphaproteobacteria (Curson et al., 2017). The basis of the need for DMSP is not entirely understood. Several physiological functions for DMSP in phytoplankton and green algae have been demonstrated, including roles as an osmolyte, antioxidant, predator deterrent, and cryoprotectant (Kirst et al., 1990; Karsten et al., 1996; Wolfe and Steinke, 1997; Sunda et al., 2002). At present, each of the proposed pathways for DMSP biosynthesis begins with methionine, although subsequent steps vary (**Figure 1**). The pathways proposed for phytoplankton, algae, corals, and perhaps the DMSP-producing Alphaproteobacteria share similar reactions and intermediates which differ distinctly from those predicted in the coastal angiosperms (Hanson et al., 1994; Gage et al., 1997; Kocsis et al., 1998; Lyon et al., 2011; Curson et al., 2017). These variations indicate that the ability to synthesize DMSP has evolved at least twice (Stefels, 2000).

**FIGURE 2:**
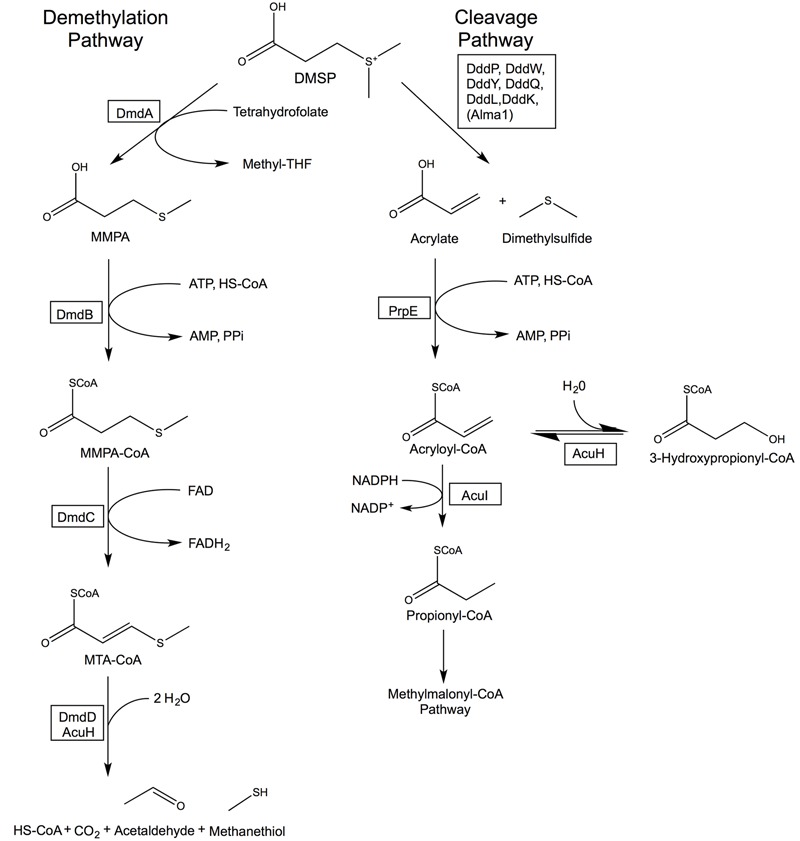
**Bacterial DMSP demethylation and cleavage pathways.** The DMSP demethylation pathway is catalyzed by the DMSP demethylase (DmdA), MMPA-CoA ligase (DmdB), MMPA-CoA dehydrogenase (DmdC), and either the MTA-CoA hydratase (DmdD) or acrylate utilization hydratase (AcuH). The cleavage pathway is catalyzed by a DMSP lyase (DddP, DddW, DddY, DddQ, DddL, DddK, or the algal Alma1), an acrylate-CoA ligase (PrpE), and an acryloyl-CoA reductase (AcuI). AcuH catalyzes a side reaction forming 3-hydroxypropionyl-CoA in the cleavage pathway. Revised from Reisch et al. (2011b, 2013).

**FIGURE 3:**
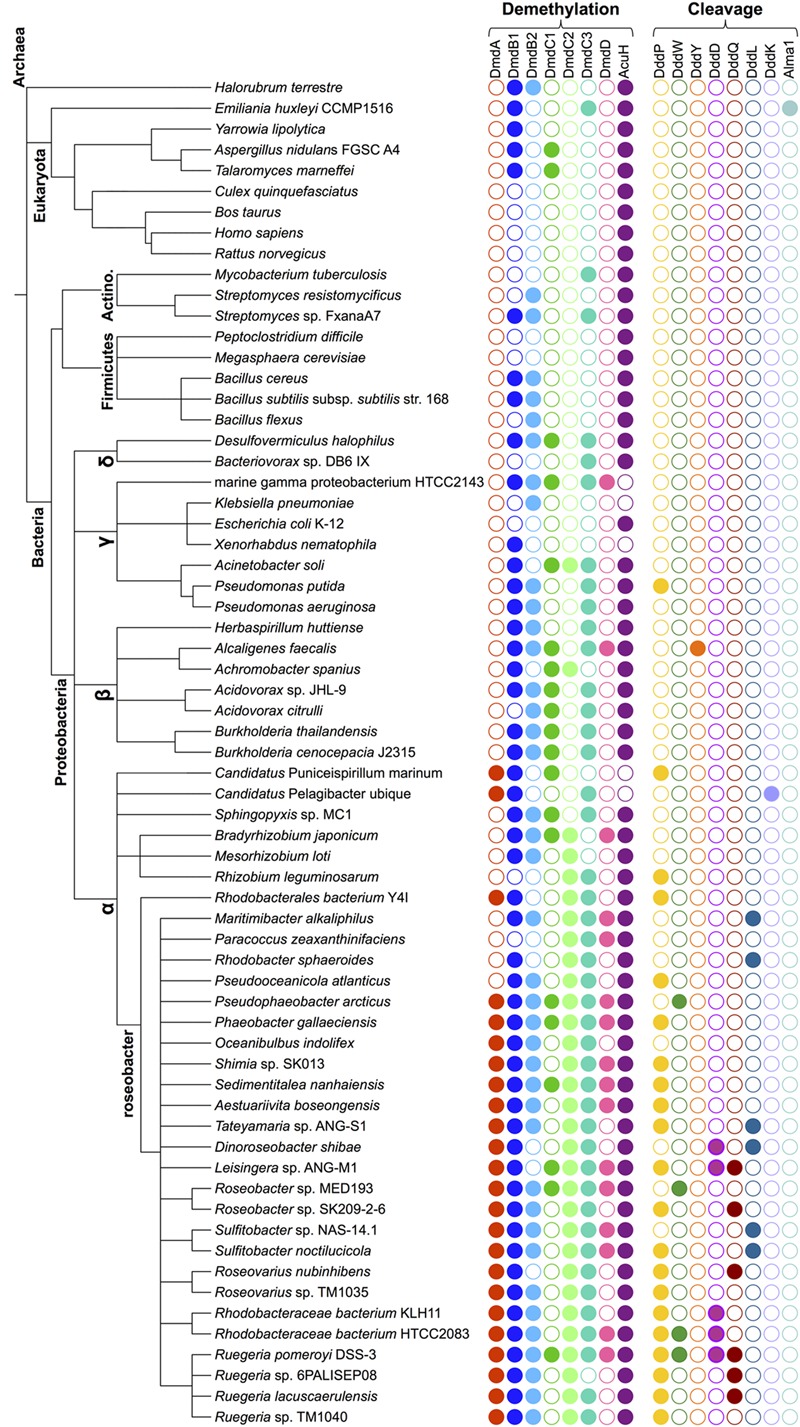
**Phylogenetic species tree representing the diversity of organisms that possess enzymes from the DMSP demethylation pathway and the DMSP lyases.** The relatedness of representative lineages is indicated schematically on the left. Colored filled circles represent the presence of the indicated protein-encoding gene. Protein designations and query sequences are as follows: From *R. pomeroyi*, DmdA (SPO1913); DmdB1 (SPO0677); DmdB2 (SPO2045); DmdC1 (SPO3805); DmdC2 (SPO0298); DmdC3 (SPO2915); DmdD (SPO3804); and AcuH (SL1157_0807) from *R. lacuscaerulensis.* DMSP lyase protein designations and query sequences are *R. pomeroyi* DddP (SPO2299), DddW (SPO0453), DddQ (SPO1596), DddD (SPO1703); *A. faecalis* DddY (ADT64689.1); *R. sphaeroides* DddL (RSP1433), *P. ubique* DddK (SAR11_0394); *E. huxleyi* Alma1 (XP_005784450). The e value cut off used in all cases was < e 10^-70^ with the exception of DddW (cut off of < e 10^-40^) and DddQ (cut off of < e 10^-30^). See **Figure 2** for the names of the enzymes.

Bacteria may metabolize DMSP via two pathways, the cleavage or the demethylation pathway (**Figure 2**). The cleavage pathway results in the formation of DMS, while the demethylation pathway produces methanethiol (MeSH). The DMSP demethylation and cleavage pathway enzymes are hypothesized to be adapted versions of enzymes that were already contained within bacterial genomes and developed in response to the availability of this substrate (Reisch et al., 2011a,b). In this review, we investigate the likely evolutionary path that led to the development of DMSP biosynthesis and subsequently the specialized DMSP catabolic pathways. The members of the Alphaproteobacteria, specifically members of the Roseobacter clade, appear to be uniquely adapted to utilize this valuable source of reduced carbon and sulfur. Bacteria within the Roseobacter and SAR11 clades possess enzymes that specifically and efficiently catalyze reactions of the demethylation pathway (Reisch et al., 2008, 2011b; Curson et al., 2011b; Tan et al., 2013; Bullock et al., 2014; Johnston et al., 2016; Sun et al., 2016). Bacteria are also responsible for the majority of DMSP catabolism via the cleavage pathway (**Figure 2**). There is additional evidence suggesting the use of DMSP as an osmolyte and antioxidant in marine bacteria (Kiene et al., 2000; Simo et al., 2002; Lesser, 2006; Reisch et al., 2011b; Salgado et al., 2014). Many microorganisms encode enzymes that share a great deal of similarity to the demethylation pathway enzymes (**Figure 3**), demonstrating their adaptability and plasticity. The many roles of DMSP may have helped to drive the adaptation of existing enzymes for DMSP metabolism.

## Evolution of Modern Phytoplankton

The first photosynthetic eukaryotes developed as the result of the acquisition of a cyanobacterium endosymbiont by a eukaryotic host, creating a membrane bound plastid (Bhattacharya and Medlin, 1998; Palmer, 2003; Yoon et al., 2004). Further diversification led to the formation of three clades from this original photosynthetic eukaryote, the green algae (green plastid lineage), the red algae (red plastid lineage), and the microbial algae glaucophytes (Delwiche, 1999). These lineages are distinguished by the chlorophyll present in their plastids. All the plastids contain chlorophyll a, but the green plastids also contain chlorophyll b, and the red plastids contain phycobilin (Keeling, 2010). Members of the Charophyta branch of the green plastid lineage colonized the land approximately 430 million year ago (mya). The Chlorophyta branch evolved into the green algae species seen today, including the Euglenoids and Chlorarachniophytes (Sanderson, 2003; Lewis and McCourt, 2004; McCourt et al., 2004). Meanwhile, today’s marine phytoplankton are largely descended from the red plastid lineage. The red plastid lineage phytoplankton, including coccolithophores, diatoms, and most dinoflagellates, first began to increase in abundance after the end-Permian extinction about 250 mya (Falkowski et al., 2004a,b).

The coccolithophores and dinoflagellates both began appearing in the fossil record about 250 mya in the Mesozoic period, while diatoms first appeared during the Early Cretaceous. All three groups saw extensive subsequent diversification in the Mesozoic period (250–65 mya) (Harwood and Nikolaev, 1995; Moldowan et al., 1996; Stover et al., 1996; Moldowan and Talyzina, 1998; Moldowan and Jacobson, 2000; Bown et al., 2004). The red lineage first began to proliferate in the benthic coastal regions, which were the first consistently oxic marine habitats. The breakup of Pangea increased sea levels and the total length of coastal area available for phytoplankton to colonize. This event also allowed nutrients that had been locked in the interior portions of continents to reach coastal waters (Vail et al., 1977; Haq et al., 1987). Changes in ocean redox chemistry from more reducing conditions that favored the green plastid lineage prior to the end-Permian extinction to the higher oxidation states of the Mesozoic ocean further contributed to the success of the red plastid lineage (Whitfield, 2001). Quigg et al. (2003) present evidence for the role of trace element availability in the proliferation of the red plastid lineage based on differences in the trace element composition between members of the red and green plastid lineages (Quigg et al., 2003). Members of the green plastid lineage have much higher requirements for iron, zinc, and copper while members of the red plastid lineage have high requirements for manganese, cobalt, and cadmium. It has been predicted that these differences in trace element requirements reflect differences in green vs. red plastid biochemistry (Quigg et al., 2003; Falkowski et al., 2004a).

The dominance of the red plastid lineage is such that all but one of the eight major taxa of eukaryotic phytoplankton in the present day oceans contains the red plastid (Falkowski et al., 2004a). The diversity of the red plastid lineage also greatly expanded as a result of secondary and tertiary endosymbiotic events, which are evident from the presence of multiple membranes surrounding some plastids of modern day phytoplankton. These events involved the engulfment of an algal cell by another eukaryote via endocytosis (Delwiche, 1999; Archibald and Keeling, 2002; Palmer, 2003; Keeling, 2010). The majority of the phytoplankton present today are the result of secondary and sometimes tertiary endosymbiotic events (Archibald, 2009). Today’s phytoplankton play key roles in global nutrient cycles and particularly in the global sulfur cycle as producers of DMSP and DMS (Lovelock et al., 1972).

## Phytoplankton and DMSP

Marine phytoplankton and algae live in an environment that is continually changing based on shifts in ocean currents. Living in this dynamic environment requires that these organisms adapt continually to varying temperatures, light, and nutrient availability (Cortes et al., 2001; Wagner et al., 2005; Allen et al., 2006). These changes may have been even more extreme in the Paleozoic and Mesozoic oceans. Abiotic forces have been found to have a large impact on the population variability of *Emiliania huxleyi* and *Florisphaera profunda* (Cortes et al., 2001). Phytoplankton in general, however, adapt quickly and relatively readily to environmental changes due to their rapid cell division rates and large population sizes (Simo, 2001; Allen, 2005; Wagner et al., 2005; Allen et al., 2006).

One specific adaptation that may help phytoplankton deal with their ever changing environment is the ability to synthesize and utilize DMSP and DMS. DMSP makes up about 90% of the reduced sulfur found in algae, but much about the regulation of its biosynthesis and uptake is still not well understood (Gage et al., 1997). Nevertheless, many of the proposed roles for these compounds would be beneficial to phytoplankton trying to survive in an ever-changing environment. DMSP is proposed to have roles as an osmolyte (Kirst et al., 1990), an antioxidant (Sunda et al., 2002), and as a means of balancing excess cellular energy (Stefels, 2000; Allen, 2005). Additionally, polar diatoms and algae are thought to produce DMSP as a cryoprotectant (Karsten et al., 1996). This hypothesis is supported by the higher levels of DMSP in sea ice diatoms compared with those from more temperate climates (Lyon et al., 2011; Kettles et al., 2014). DMSP is also a predator/grazing deterrent owing to its cleavage to acrylate (Stefels, 2000). New studies of the coral genus *Acropora* have generated still more uses for DMSP. Reef building coral juveniles increase DMSP production when subject to thermal stress and may also use DMSP as a bacterial signaling molecule, attracting particular microbial communities that are necessary for coral health (Raina et al., 2013).

The role of DMSP and DMS as antioxidants could be particularly useful for phytoplankton as plastids are typically hyperoxic and produce reactive oxygen species (ROS) during oxygenic photosynthesis. Other stresses such as exposure to ultraviolet radiation (UVR) and thermal stress can further increase ROS production (Asada and Takahashi, 1987; Fridovich, 1998; Lesser, 2006). The production of ROS by plastids might explain why DMSP and DMS production are observed in both phytoplankton and land plants. There is also evidence to suggest that the final step of DMSP synthesis in the flowering plant *W. biflora* takes place in the plastid (chloroplast) (Trossat et al., 1996). DMSP, DMS, and acrylate are all able to quench HO^∙^ radicals, although acrylate and DMS are more efficient than DMSP. The resultant product of HO^∙^ quenching is dimethylsulfoxide (DMSO), which subsequently reacts with additional HO^∙^ radicals to form methane sulfinic acid and then methane sulfonic acid. In contrast to DMSP and acrylate, DMS is uncharged and can diffuse through biological membranes, acting as an antioxidant nearly anywhere in the cell (Sunda et al., 2002; Lesser, 2006; Husband et al., 2012).

Another impetus for the production of DMSP may be the need for an osmolyte that does not contain nitrogen. Nitrogen is often limiting in ocean surface waters, which may in turn limit the production of the nitrogen-containing osmolyte glycine betaine. Ito et al. (2011) observed that under conditions where sulfate limited growth of the marine algae *Ulva pertusa*, the sulfur from methionine was used primarily for the synthesis of *S*-adenosyl methionine and methionyl-tRNA, rather than for DMSP synthesis. However, when the salinity and abundance of sulfate increased; the sulfur from methionine was increasingly used for DMSP biosynthesis, and the intracellular DMSP levels increased (Ito et al., 2011).

One additional hypothesis for the origin of DMSP biosynthesis proposes that it developed as a means of dispelling excess energy, carbon and reducing equivalents when growth becomes unbalanced due to nutrient limitation (Stefels, 2000). Rapid changes in the ocean environment can require phytoplankton to have an equally rapid response to imbalances between photosynthesis and growth (Allen, 2005; Wagner et al., 2005; Allen et al., 2006). Since photon capture cannot be quickly stopped, production of nitrogen or phosphorous poor molecules when growth is limited by these nutrients is a means of consuming extra carbon, energy, and reducing equivalents that cannot be used for protein biosynthesis or cell division (Stefels, 2000; Simo, 2001; Allen, 2005). Further, the continued production of DMSP may also serve to regenerate and redistribute nitrogen for the production of new amino acids and to stimulate continued sulfate assimilation by keeping the cellular concentration of methionine and cysteine low (Gage et al., 1997; Stefels, 2000). Thus, DMSP may have originally been produced as a means of dissipating excess energy and carbon and was then adapted for other functions.

## Synthesis of DMSP by Marine Phytoplankton and Algae

The main producers of DMSP are phytoplankton, mostly in the classes Dinophyceae (dinoflagellates) and the Prymnesiophycaea (which includes the coccolithophores). Certain members of the Chryosphyceae and Bacillariophyceae (diatoms) can also produce DMSP (Keller, 1989). DMSP likely first became abundant in ocean environments about 250 mya in conjunction with the increasing abundance of dinoflagellates and coccolithophores. Based on a comparison of literature reports for 95 DMSP-producing species (**Table 1**), it was determined that dinoflagellates produced the highest amounts of DMSP, with intracellular levels ranging from 0.00011 to 14.7 pmol/cell (Keller, 1989; Wiesemeier and Pohnert, 2007; Caruana, 2010; Caruana and Malin, 2014). In particular, *Alexandrium minutum* and *Protoperidinium pellucidum* produced 14.2 and 14.7 pmol DMSP/cell, respectively. Diatoms have intracellular DMSP levels ranging from 0.0006 to 0.257 pmol/cell, while haptophytes (coccolithophores) contained from 0.00037 to 0.148 pmol DMSP/cell (Keller, 1989; Caruana and Malin, 2014). DMSP production is less common among the higher plants, although it has been observed in *Spartina* species (Kocsis et al., 1998), certain sugarcanes (Paquet et al., 1994), and the flowering plant *W. biflora* (Hanson et al., 1994; James et al., 1995). DMSP production has also been observed in members of the coral genus *Acropora* in the absence of their algal endosymbiont *Symbiodinium*, also a known DMSP producer (Raina et al., 2013). Thus, while DMSP is widely distributed in a large number of phototrophs, only a few groups produce very high amounts, and it is likely that DMSP only became widely available as a nutrient in marine environments following the evolution of these groups.

**Table 1:** Levels of DMSP production for different phytoplankton groups^a^.

Taxonomic group	Number of species examined	Concentration of DMSP (median pmol/cell)	Range (pmol/cell)
Dinoflagellates	40	0.1725	0.00011–14.7
Diatoms	15	0.00745	0.0006–0.257
Chlorophytes	9	0.001	0.00015–0.012
Golden algae	4	8.36 × 10^-04^	0.000149–0.02
Haptophytes	15	0.0158	0.000373–0.148
Cryptophytes	1	0.0213	NA^b^
Rhodophyta	1	0.00231	NA
Cyanobacteria	1	8.94 × 10^-06^	7.45–10.4 × 10^-6^
Coral	6	0.0826	0.021–3.331
Zooxanthellae	4	0.14	0.048–0.285

*^a^The data in this table was produced by synthesizing data from the following sources: (Keller, 1989; Corn et al., 1996; Yoch, 2002; Wiesemeier and Pohnert, 2007; Breckels et al., 2010; Caruana, 2010; Caruana and Malin, 2014). ^b^Not applicable.*

Little is understood about the biosynthetic pathways for DMSP in marine phytoplankton and corals. The first complete DMSP biosynthetic pathways were described in the green algae *U. intestinalis* (Gage et al., 1997), the marine cordgrass *S. alterniflora* (Kocsis et al., 1998), and coastal plant *W. biflora* (Hanson et al., 1994; James et al., 1995) (**Figure 1**). Each pathway identified thus far begins with methionine and includes a deamination reaction, supporting the hypothesis that DMSP biosynthesis is used by these organisms to regenerate nitrogen from methionine. The DMSP biosynthetic pathways of *S. alterniflora* and *W. biflora* are more similar to each other than they are to the pathway in *U. intestinalis*, suggesting that the plant pathways evolved independently from those in marine algae, corals, and phytoplankton. If true, this would indicate that there was selective pressure for the evolution of DMSP biosynthetic pathways even in very different organisms.

The DMSP biosynthetic pathways of the major producers in the marine environment are still largely unknown, but they are likely similar to the pathway described in *U. intestinalis*. The *U. intestinalis* pathway begins with methionine and utilizes an aminotransferase, a NADPH-linked reductase, a methyltransferase, and an oxidative decarboxylase to produce DMSP (Gage et al., 1997; Summers et al., 1998). The commitment step is hypothesized to be the third step, the conversion of 4-methylthio-2-hydroxybutyrate (MTHB) to 4-dimethylsulfonio-2-hydroxybutyrate (DMSHB) by a methyltransferase (**Figure 1**). The key intermediate DMSHB has been identified in *U. intestinalis, U. pertusa, E. huxleyi, Tetraselmis* sp., and *Melosira nummuliodes*, indicating that this pathway is present in a range of phytoplankton (Gage et al., 1997; Stefels, 2000; Ito et al., 2011). Lyon et al. (2011) identified candidate proteins and genes for this four-step pathway in the sea-ice diatom *Fragilariopsis cylindrus.* Proteins from the same enzyme classes proposed in the *U. intestinalis* pathway were more abundant when *F. cylindrus* was exposed to conditions that increased DMSP production. However, the activities of these proteins still need to be verified (Lyon et al., 2011). Orthologs for the genes encoding a NADPH-reductase and an AdoMet-dependent methyltransferase have also been found in the corals *Acropora millepora* and *Acropora digitifera* and in the coral dinoflagellate symbiont *Symbiodinium*, all known DMSP producers. Based on the collective data, Raina et al. (2013) hypothesized that the enzymes of the DMSP biosynthetic pathway are conserved between diatoms, alveolates, green algae, and corals. Interestingly, a study of the diatom *Thalassiosira pseudonana* did not identify any of the same proteins proposed for the *F. cylindrus* biosynthetic pathway under conditions that increased intracellular DMSP levels, suggesting that it may contain an alternative pathway (Kettles et al., 2014).

A recent study has reported the biosynthesis of DMSP by marine bacteria. DMSP production was observed from *Oceanicola batsensis* HTCC2597, *Pelagibaca bermudensis* HTCC2601, *Sediminimonas qiaohouensis* DSM21189, *Amorphus coralli* DSM18348, *Sagittula stellata* E-37, *Labrenzia aggregata* LZB033, *Labrenzia aggregata* IAM12614, and *Thalassobaculum salexigens* DSM19539 (Curson et al., 2017). DMSP biosynthesis in marine bacteria proceeds in a similar manner to that observed in algae and phytoplankton, via the methionine transamination based pathway. A methyltransferase gene, *dysB*, was identified in marine Alphaproteobacteria and appears to be the key enzyme for DMSP biosynthesis in these microorganisms. When *dysB* was cloned into the non-DMSP producer *Rhizobium leguminosarum*, the ability to synthesize DMSP was conferred. Thus, the addition of a single gene, in certain cases, is sufficient to enable the production of DMSP. Further, *dysB* expression from *L. aggregata* LZB033 is up-regulated during increased salinity, nitrogen limitation, and at low temperatures, conditions already predicted to stimulate DMSP production in marine phytoplankton and algae. Selective pressures, like changes in salinity or nitrogen limitation, could result in the acquisition of *dysB* by marine bacteria to enable DMSP biosynthesis and gain a competitive advantage in their environment (Curson et al., 2017).

## DMSP Cleavage by Marine Phytoplankton

While the demethylation pathway appears to be unique to marine bacteria, several marine phytoplankton lyse DMSP into DMS. Multiple studies have reported significant DMSP lyase activity within phytoplankton blooms and among individual phytoplankton, including *Phaeocystis* sp., *Heterocapsa triquetra, Scrippsiella trochoidea*, and several *Symbiodinium* strains (Stefels et al., 1995; Niki et al., 1997, 2000; Yoch, 2002). To date, while several marine phytoplankton have been observed to produce DMS from DMSP, the genes responsible for this activity have not been identified in most cases. It has been known for many years that *E. huxleyi* cleaves DMSP into DMS and acrylate (Yoch, 2002), but only recently was the responsible gene, *Alma1*, identified (Alcolombri et al., 2015). Alma1 is a member of the aspartate racemase superfamily. Based on sequence similarity, Alma1 and its paralogs from *E. huxleyi* are present in a wide range of phytoplankton as well as certain bacteria, highlighting the diversity of this protein (Yost and Mitchelmore, 2009; Alcolombri et al., 2015). There are seven *Alma1* paralogs within the *E. huxleyi* genome. Alma1 paralogs from *E. huxleyi, Phaeocystis Antarctica, A. millepora* (coral), and *Symbiodinium* sp. were synthesized and tested for activity toward DMSP. Of those tested, however, only one *E. huxleyi* paralog, *Alma2*, and a *Symbiodinium* paralog had DMSP lyase activity, indicating that there is still much to learn about the phytoplankton DMSP lyases (Alcolombri et al., 2015).

## Bacterial Pathways for DMSP Metabolism

Marine bacteria have developed many uses for DMSP, from a source of reduced sulfur and carbon (Kiene et al., 1999, 2000), to use as an osmolyte (Sunda et al., 2002; Salgado et al., 2014), and potentially a cryoprotectant (Karsten et al., 1996). The details of the bacterial catabolism of DMSP have only recently come to light (**Figure 2**). The characterization of the enzymes involved in the DMSP demethylation pathway as well as the identification of several DMSP lyases from the DMSP cleavage pathway have provided new insights into the evolution of these enzymatic activities. Some of the enzymes of the demethylation pathway have likely roots in fatty acid ß-oxidation (Reisch et al., 2011a,b; Bullock et al., 2014). The DMSP lyases are widely distributed and varied in sequence, structure, and activity (Curson et al., 2011b; Johnston et al., 2016). Many of the enzymes involved in the microbial DMSP catabolic pathways are widespread, particularly among the Proteobacteria (**Figure 3**). Even those Roseobacters with reduced genomes, such as the lineages SAG-O19, DC5-80-3, and NAC11-7, have been found to encode *dmdA* and at least one DMSP lyase (Zhang et al., 2016). Presumably, the relatively modern evolution of phytoplankton producing high levels of DMSP provided the impetus for developing and maintaining these functions. To learn more about how the degradation pathways evolved, the structural and functional characteristics of the DMSP catabolic enzymes were examined to posit how they may have been adapted from existing enzymes.

## Enzymatic Cleavage of DMSP

The enzymatic cleavage of DMSP produces DMS and acrylate. To date, eight DMSP lyases have been identified (**Table 2**). The lyases were recently reviewed (Johnston et al., 2016). Except DddD which produces 3-hydroxypropionate, these enzymes all carry out the same reaction to form DMS and acrylate despite differing drastically in sequence and size (Todd et al., 2010; Curson et al., 2011b). Based upon a survey of lyase encoding genes in representative genomes of marine bacteria, *dddP* is the most widely distributed (**Figure 3**). However, *dddD, dddW, dddQ*, and *dddL* are also relatively common. In contrast, *dddY, dddK* and Alma1 are rare in marine bacteria. There are now several reports of DMSP lyase activity being induced by the presence of DMSP. In *Ruegeria pomeroyi* DSS-3 and *Roseovarius nubinhibens, dddP* and *dddQ* expression was induced when cells were pre-grown with DMSP as compared to cells not exposed to DMSP. Likewise, expression of *dddY* increased following growth of *Alcaligenes faecalis* with DMSP (Todd et al., 2007, 2009). Further, a field study in Monterey Bay, California, found that expression of *dddP* increased during mixed-community DMSP-producing phytoplankton blooms (Varaljay et al., 2015). Expression of *R. pomeroyi dddW* also increased after exposure to DMSP in growth medium (Todd et al., 2012b). These observations are consistent with a role of these enzymes in DMSP cleavage.

**Table 2:** Identified DMSP lyases and their *K*_m_ for DMSP.

Protein	Organism	*K*_m_ for DMSP	Reference
DddY	*A. faecalis, D. acrylicus*	1.4 mM, 0.4 mM	de Souza and Yoch, 1995; van der Maarel et al., 1996; Curson et al., 2011a
DddD	*Marinomonas*	>40 mM^a^	Todd et al., 2007; Alcolombri et al., 2014
DddL	*Sulfitobacter* EE-36	ND^b^	Curson et al., 2008
DddP	*R. lacuscaerulensis, R. nubinhibens*	17 mM, 14 mM	Todd et al., 2009; Kirkwood et al., 2010; Wang et al., 2015
DddW	*R. pomeroyi*	8.7 mM	Todd et al., 2012b; Brummett et al., 2015
DddQ	*R. lacuscaerulensis*	22 mM	Todd et al., 2011; Li et al., 2013
DddK	*P. ubique* HTCC1062	82 mM	Sun et al., 2016
Alma1	*E. huxleyi*	9.0 mM	Alcolombri et al., 2015

*^a^Saturation was not observed at 40 mM. ^b^No data.*

Evidence for the physiological relevance of the two best studied lyases, DddP and DddQ, has been mounting. The *dddP* and *dddQ* genes are the most abundant of the bacterial DMSP lyase genes in the marine metagenome as determined by the Global Ocean Sampling Expedition (GOS) (Rusch et al., 2007; Todd et al., 2011). The role of DddP and DddQ from *R. pomeroyi* DSS-3 and *R. nubinhibens* in DMSP cleavage has been clearly demonstrated. Studies using ^14^C or ^13^C labeled DMSP show that *Escherichia coli* extracts expressing *dddP* and *dddQ* are able to produce DMS and acrylate from DMSP (Kirkwood et al., 2010; Todd et al., 2011). Additionally, *dddP* and *dddQ* mutants in *R. pomeroyi* produce significantly less DMS when compared with wild-type cells, 50% less in the case of *dddP* and 97% less in the case of *dddQ*. A *dddQ* mutant from *R. nubinhibens* produced 20% less DMS from DMSP, while a *dddP* mutant produced only 10% of the wild-type levels (Todd et al., 2009, 2011; Kirkwood et al., 2010).

The structures of the DMSP lyases provide insights into their evolutionary roots. The crystal structures of DddP and DddQ from *Ruegeria lacuscaerulensis* and DddP from *Roseobacter denitrificans* have been solved (Li et al., 2013; Wang et al., 2015). Data gathered from the available structures suggests that subtle changes in the active sites of these lyases make sulfur containing substrates, like DMSP, the preferred substrates for these enzymes. The sequence and structure of DddP most closely resembles that of M24 peptidase. Typically, an M24 peptidase hydrolyzes C-N bonds. DddP, however, cleaves C-S bonds (Todd et al., 2009; Wang et al., 2015). Wang and coworkers expressed the recombinant *R. lacuscaerulensis dddP* in *E. coli* and found it displayed no measurable activity toward the M24 peptidase substrate valine-proline, but it did exhibit DMSP lyase activity, producing acrylate and DMS (Wang et al., 2015). DddP is a homodimeric protein in which one monomer has a metal center containing Fe, while the other monomer generally contains Fe, but may also contain Ni, Zn, or Cu instead (Hehemann et al., 2014; Wang et al., 2015). The explanation for the change in substrate preference and activity appears to be due to the change of the active ion from Co or Mn coordinated by five residues in the M24 peptidases to Fe coordinated by six residues in DddP. The two metal ions in DddP are coordinated with three aspartates, two glutamates, and a histidine residue, which are conserved in the known functional DddPs (Hehemann et al., 2014; Wang et al., 2015). The substitution of any of the active site residues for alanine in DddP results in the elimination of DMSP lyase activity, indicating that all six are necessary for activity (Kirkwood et al., 2010; Wang et al., 2015). Additionally, two conserved histidine residues in M24 peptidases that help to bind and stabilize substrates are exchanged for aspartate and phenylalanine in DddP (Hehemann et al., 2014; Wang et al., 2015). Wang et al. (2015) suggest that this change abolishes the peptidase activity of DddP and allows the active site aspartate to act as a nucleophilic base for DMSP cleavage (Wang et al., 2015). It is further proposed that DddP is a case of divergent evolution from the M24 peptidases as DddPs cluster in a separate clade in phylogenetic analyses. In support of this hypothesis, the M24 peptidase conserved C-domain has up to 31% sequence identity with the C-domain of the *R. lacuscaerulensis* DddP. The N-domain of DddP, by contrast is structurally different than the N-domains of M24 peptidases and allows for the formation of a compact dimer and a smaller catalytic cavity for DMSP binding (Wang et al., 2015). In conclusion, DddP appears to have acquired specific adaptations for DMSP lyase activity, supporting the assertion that this is its major role.

A structure for DddQ from *R. lacuscaerulensis* has recently been solved (Li et al., 2013). DddQ is one of the cupin motif containing DMSP lyases, along with DddW and DddL (Curson et al., 2011b; Johnston et al., 2016). DddQs have been identified in a number of Roseobacters, but they display substantial amino acid sequence variation, even when multiple copies are present in the same organism (Todd et al., 2011). Despite this variation, certain amino acids in the cupin motifs, two histidines and a glutamate in cupin motif 1 and a histidine in cupin motif 2 are conserved in DddQ, DddW, and DddL. In addition to these conserved amino acids, two tyrosines in motif 1 are highly conserved in all the cupin protein DMSP lyases but not among other cupin proteins. These conserved active site residues are predicted to play a role in DMSP cleavage as substitution at any of these residues decreased activity toward DMSP (Li et al., 2013).

The formation of DMS and acrylate from DMSP is proposed to be the result of a β-elimination reaction (Li et al., 2013; Wang et al., 2015). The DMSP lyases appear to have developed different catalytic mechanisms for carrying out the same reaction, indicting separate evolutionary paths to this activity. DddP is proposed to implement an ion shift. When DMSP enters the active site, a moveable Fe binds to the carboxyl group of DMSP, stabilizing the molecule in the active site, while two other conserved residues, tryptophan and tyrosine, bind to the sulfur in DMSP. This orientation allows for the abstraction of a proton by aspartate from the alpha carbon of the DMSP carboxyl group, cleavage of the C-S bond, and the subsequent formation of a double bond between the alpha and beta carbons of DMSP to produce acrylate (Hehemann et al., 2014; Wang et al., 2015). In DddQ, it has been proposed that binding of DMSP to the metal cofactor causes a conserved tyrosine residue to shift closer to the DMSP molecule. This shift allows the oxygen atom of one of the conserved tyrosine residues to interact with the alpha carbon of DMSP. The resultant conformational change enables the abstraction of a proton from the DMSP carboxyl group by the oxygen atom of tyrosine (Li et al., 2013). The algal DMSP lyase, Alma1, is proposed to function in a similar manner, abstracting a proton from the carbon adjacent to the carboxylate to cause β-elimination and the subsequent release of DMS and acrylate (Alcolombri et al., 2015). Further investigations into the structures and mechanisms of the other DMSP lyases, like the algal Alma1 or DddY, may yield still more variability in reaction mechanisms.

DddY from *A. faecalis* M3A was the first identified DMSP lyase (de Souza and Yoch, 1995). It is the only DMSP lyase that is a periplasmic protein and has no similarity to any other enzyme of known function. DddYs have been identified in *A. faecalis* M3A and *Desulfovibrio acrylicus*, as well as in several *Shewanella* species and *Arcobacter nitrofigilis* DSM7299. DMS production from DMSP was observed in *Shewanella halifaxensis* HAW-EB4, *Shewanella putrefaciens* CN-32, and *A. nitrofigilis* DSM7299 (Curson et al., 2011a). *S. halifaxensis* and *S. putrefaciens* are found in marine sediments and shale sandstone, respectively, while *A. nitrofigilis* can be found in sediment around *Spartina* roots. It is likely that *dddY* was spread via horizontal gene transfer (HGT) among these distantly related bacteria. In addition to *dddY, A. faecalis* also has acrylate utilization (*acu)* genes that resemble those used for DMSP and acrylate metabolism in other DMSP-utilizing bacteria (Curson et al., 2011a). More in depth studies of DddY have not been undertaken.

Despite convincing evidence for the physiological role of the DMSP lyases, the affinities for DMSP of the currently known lyases are lower than expected for a natural substrate, displaying *K*_m_s for DMSP in the millimolar range (**Table 2**) (Johnston et al., 2016). The *K*_m_s for the most widely distributed lyases, DddP and DddQ, are among the highest (Rusch et al., 2007; Todd et al., 2011). The lowest *K*_m_ for DMSP observed thus far is for DddY. The DddYs from *A. faecalis* and *D. acrylicus* have *K*_m_s for DMSP of 1.4 and 0.4 mM, respectively (**Table 2**) (de Souza and Yoch, 1996; van der Maarel et al., 1996). Both of these organisms are found in coastal marine sediments and likely obtain DMSP from *Spartina* spp. (Curson et al., 2011a). High *K*_m_ values for DMSP are also shared with the DMSP demethylases (see DmdA below), which is the first committed step of the demethylation pathway. Thus, the low affinities of the lyases may simply reflect the requirement for high intracellular concentrations of DMSP to initiate its metabolism. If DMSP serves as an osmolyte in bacterioplankton, cells should maintain high concentrations in the cell. For instance, during growth on DMSP, a concentration of 70 mM has been observed in *R. pomeroyi*. Under these conditions, low *K*_m_s for DMSP are not necessary for DMSP lyases to function effectively *in vivo* (Reisch et al., 2008). Concentrations of DMSP in ocean surface waters range from less than 1 nM in the open ocean to micromolar levels within phytoplankton blooms (van Duyl et al., 1998). Senescence and autolysis of DMSP producers like *Spartina* or phytoplankton can also produce microenvironments with high concentrations of DMSP (de Souza and Yoch, 1995). Provided a bacterium has the necessary transporters for the uptake of DMSP, intracellular concentrations of DMSP have the potential to reach to millimolar levels (Kiene, 1998; Kiene and Williams, 1998; Kiene et al., 1998; Todd et al., 2010).

In conclusion, the sequence and structural variability of the DMSP lyases that have been identified so far indicates that they likely evolved independently. For this to happen, the new activity must be readily acquired in evolution from multiple ancestral enzymes, Moreover, there must be strong selective pressures to maintain this function in very different groups of organisms. In addition, some bacteria contain multiple DMSP lyases, and it is possible that their physiological functions are somewhat different. This would allow cells to maintain lyases with the same catalytic activity but different regulatory or other functional properties.

## Bacterial Demethylation of DMSP

The DMSP demethylation pathway consists of a series of reactions that convert DMSP into methanethiol (MeSH), HS-CoA, CO_2_, and acetaldehyde (Reisch et al., 2011a,b). While DMS production from phytoplankton has been observed, there is no indication that these organisms possess the demethylation pathway. Instead, the demethylation pathway is restricted to the Alphaproteobacteria (**Figure 3**). Based on the current evidence, it seems likely that the individual steps of the demethylation pathway may have evolved independently.

## DmdA: An Adapted Glycine Cleavage T-Protein

The initial step of the demethylation pathway is mediated by the DMSP demethylase DmdA (**Figure 2**). This step also commits DMSP to the demethylation pathway because demethylation precludes the formation of DMS (Howard et al., 2006; Reisch et al., 2008, 2011b). As with the DMSP lyases, the *K*_m_s for DMSP of the two characterized DmdAs from *R. pomeroyi* and *Pelagibacter ubique* are relatively high, 5.4 and 13.2 mM, respectively. The deletion of *dmdA* from *R. pomeroyi*, however, results in a mutant incapable of producing MeSH, indicating that this gene encodes the only protein in *R. pomeroyi* able to perform this reaction (Howard et al., 2006; Reisch et al., 2008). Additionally, field measurements indicate that *dmdA* expression is upregulated during blooms of DMSP-producing phytoplankton (Varaljay et al., 2015). DmdA in *R. pomeroyi* was initially annotated as a glycine cleavage T-protein (GcvT) (Reisch et al., 2011a). However, when analyzed phylogenetically, DmdA-like proteins share sequence identity ranging from 22 to 26% with GcvT, dimethylglycine oxidase and sarcosine oxidase, but form a separate clade from known GcvTs.

**FIGURE 4:**
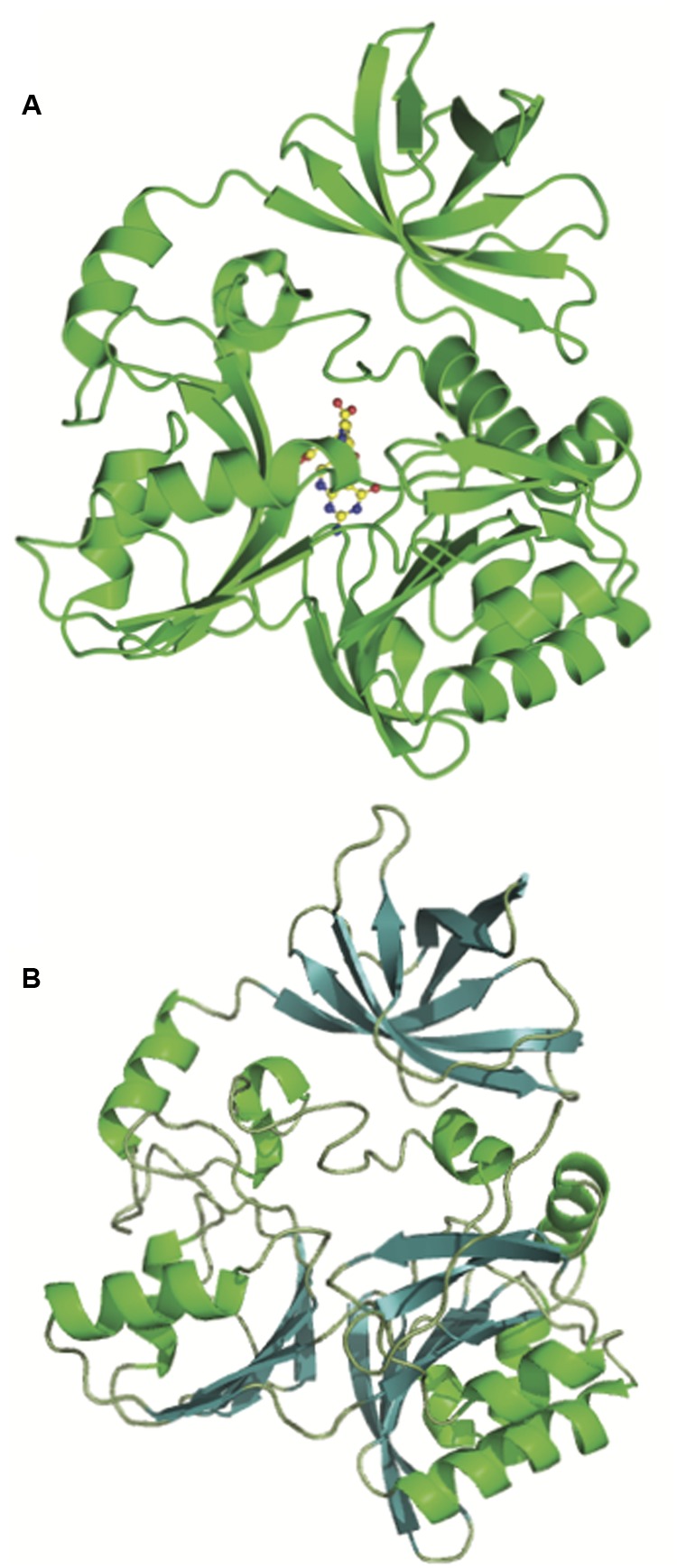
**Structural similarity of the glycine cleavage T-protein and the DMSP demethylase (DmdA).** Crystal structures of the **(A)** glycine cleavage T-protein from *Thermotoga maritima* (Lee et al., 2004) (PBD ID:1WOO) and **(B)** the DmdA monomer from *P. ubique* (Schuller et al., 2012) showing the shared tri-domain structure.

The crystal structure of DmdA from *P. ubique* provides further evidence supporting a common ancestry for DmdA and GcvT (**Figure 4**). Schuller et al. (2012) described the structure of DmdA, noting that while DmdA is structurally similar to GcvT, the low sequence similarity between the two indicated that the enzymes are evolutionarily distant (Schuller et al., 2012). Both proteins possess a very similar tri-domain structure (**Figure 4**) with the conserved residues between the proteins being mainly involved with tetrahydrofolate (THF) binding. Specifically, the residues that interact with the folate moiety and those involved in the ring stacking of THF are highly conserved (Lee et al., 2004; Reisch et al., 2008; Schuller et al., 2012). In contrast, DmdA possesses high substrate specificity for DMSP and closely related compounds, so the binding site for this substrate must differ from that of GcvT. Despite structural similarity, DmdA and GcvT are mechanistically distinct. DmdA produces 5-methyl-THF from DMSP as the result of a redox-neutral methyl transfer while GcvT coverts glycine to 5,10-methylene-THF (Howard et al., 2006; Reisch et al., 2008; Schuller et al., 2012). Small changes to the THF- binding fold in DmdA allow for hydrogen bond formation between amino acid residues in the fold and THF, enabling DmdA to carry out a redox-neutral methyl transfer to produce 5-methyl-THF. Overall, the mechanism of DmdA catalysis appears to be more similar to the *S*-adenosylmethionine SAM-dependent *N*-methyltransferases than the more closely related GcvTs (Schuller et al., 2012).

Phylogenetic analysis reveals that GcvTs and other similar proteins are nearly universally distributed among the prokaryotes, while DmdA proteins cluster separately. DmdA appears to be most prevalent among members of the Alphaproteobacteria (**Figure 3**) (Reisch et al., 2008; Moran et al., 2012). DmdA may have originally been a GcvT, but the development of a new activity and substrate preference has uniquely adapted this enzyme for DMSP metabolism (Reisch et al., 2008). Other organisms without DmdA may simply maintain DMSP as an osmolyte or utilize one of the many DMSP lyases identified so far to metabolize it.

## The Flexibility of DmdB and DmdC

Once methylmercaptopropionate (MMPA) is produced by DmdA, it is converted first to MMPA-CoA by the MMPA-CoA ligase or DmdB and then to methylthioacryloyl (MTA)-CoA by the MMPA-CoA dehydrogenase DmdC (Reisch et al., 2011a). In contrast to the narrower distribution of DmdA, DmdB and DmdC are found in up to 60% of surface ocean bacteria, assuming one copy per cell, as well as in bacteria from terrestrial and other environments (**Figure 3**) (Reisch et al., 2011b). The DmdB and DmdC enzymes characterized thus far show activity with a wide range of substrates, mostly with small to medium chain length fatty acids and their CoA derivatives (Reisch et al., 2011b; Bullock et al., 2014). These enzymes probably did not originate specifically for DMSP metabolism, potentially having been recruited from the pathways of methionine degradation and ß-fatty acid oxidation (Reisch et al., 2011a,b). The ability of DmdB and DmdC to act upon MMPA and MMPA-CoA is a demonstration of the plasticity and flexibility of these enzymes.

*R. pomeroyi* possesses more than 20 CoA ligases, but not all are predicted to have activity with MMPA. *R. pomeroyi* has two DmdB isozymes, RPO_DmdB1 and RPO_DmdB2 (**Table 3**). RPO_DmdB1 has a *K*_m_ of 0.08 mM for MMPA but even lower *K*_m_s for butyrate and propionate, 0.02 and 0.04 mM, respectively. RPO_DmdB2 has a *K*_m_ for MMPA similar to that of RPO_DmdB1, 0.07 mM, but this was the lowest *K*_m_ it displayed with any of the substrates tested (**Table 3**) (Bullock et al., 2014). There are distinct differences between the DmdB enzymes from marine and non-marine microorganisms. Particularly, only the DmdBs from marine microorganisms are inhibited by concentrations of DMSP likely to be present in the cell. The *R. pomeroyi* DmdB isozymes exhibit different regulatory mechanisms to reverse this inhibition. RPO_DmdB1 responds to changes in cellular energy charge, while RPO_DmdB2 responds to increases in MMPA concentration (Bullock et al., 2014). These regulatory mechanisms may have developed during the specialization of the DmdB isozymes for DMSP rather than fatty acid metabolism. Because they are not found in the DmdBs from terrestrial bacteria, they appear to be specific adaptations to the importance of DMSP as a nutrient for marine bacteria.

**Table 3:** Apparent kinetic constants for *R. pomeroyi* DSS-3 MMPA-CoA ligases RPO_DmdB1 and RPO_DmdB2^a^.

Substrate	Enzyme	*K*_m_ (mM)*^a^*	*k*_cat_ (s^-1^)	*k*_cat_/*K*_m_ (mM^-1^ s^-1^)
Methylmercaptopropionate	RPO_DmdB1	0.08 ± 0.02	18.7	233
	RPO_DmdB2	0.07 ± 0.02	14.9	213
Butyrate	RPO_DmdB1	0.02 ± 0.01	14.4	1031
	RPO_DmdB2	0.12 ± 0.03	7.2	71
Propionate	RPO_DmdB1	0.04 ± 0.01	10.8	271
	RPO_DmdB2	3.11 ± 1.13	3.7	1.2
Acrylate	RPO_DmdB1	0.9 ± 0.2	14.3	16
	RPO_DmdB2	5.25 ± 2.1	1.0	0.2

*^a^*K*_*m*_ (mM) is shown (±SE) from three independent experiments. *k*_*cat*_ is expressed in units of s^-*1*^ and *k*_*cat*_/*K*_*m*_ in units of mM^-*1*^ s^-*1*^. Reproduced from Bullock et al. (2014).*

Three DmdC isozymes were identified in *R. pomeroyi* and verified to have activity toward MMPA-CoA (Reisch et al., 2011b). The *K*_m_ of one of the DmdC isozymes (SPO3804; DmdC1) from *R. pomeroyi* for MMPA-CoA is low at 0.03 mM. However, lower *K*_m_s were observed for this enzyme with caproyl-CoA, valeryl-CoA, and butyrl-CoA. Thus, MMPA-CoA is not necessarily the preferred substrate for this enzyme. Instead, the substrate specificity of DmdC appears, like DmdB, to be based primarily on the length of the carbon chain of a substrate.

DmdB and DmdC isozymes are more widely distributed than DmdA, suggesting that these enzymes may be important in organisms that either metabolize only MMPA but not DMSP or possess pathways that form MMPA from substrates other than DMSP. Methionine degradation is one potential source of MMPA (Steele and Benevenga, 1979), a side reaction of the methionine salvage pathway can also produce MMPA (Sekowska et al., 2004; Albers, 2009). *Xanthomonas campestris* produces MMPA to induce bacterial blight in cassava (Perreaux et al., 1982; Ewbank and Maraite, 1990), and many plants, particularly fruiting plants, produce sulfur volatiles which closely resemble MMPA in structure (e.g., 3-methylthio-propanol, 3-methylthio-propanal, and ethyl-3-methylthio-propionate) (Gonda et al., 2013). These compounds might also be substrates of DmdB. Alternatively, the primary function of DmdB and DmdC in many bacteria may be fatty acid oxidation, and MMPA may only be an occasional substrate.

## DMSP Specific Enoyl-CoA Hydratases: DmdD and AcuH

**FIGURE 5:**
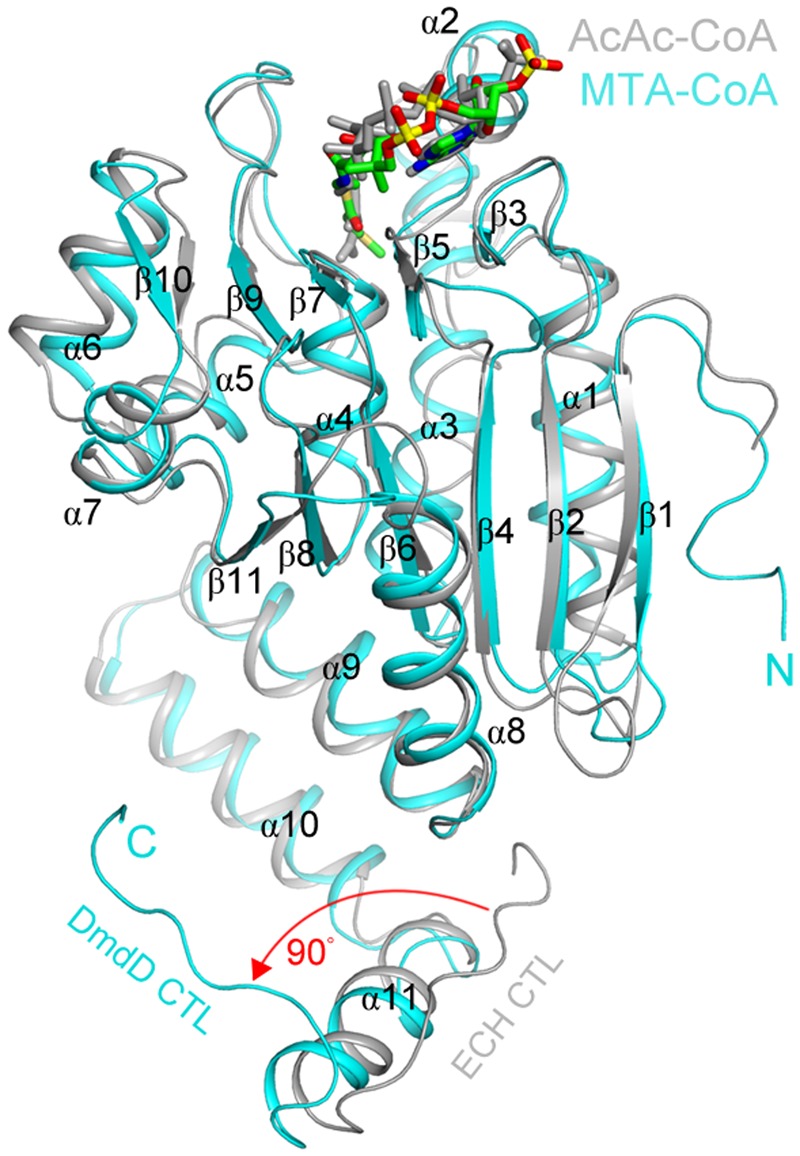
**Structural similarities of the MTA-CoA hydratase and the enoyl-CoA hydratase from rat liver.** Comparison of the structures of the *R. pomeroyi* MTA-CoA hydratase (DmdD, E121A mutant) monomer (cyan) in complex with MTA-CoA (green) and the rat liver ECH monomer (gray) in complex with acetoacetyl-CoA (gray) (Engel et al., 1996). The red arrow indicates a difference in the conformation of the C-terminal loop between the two structures. Figure reproduced from Tan et al. (2013).

DmdD, a member of the crotonase superfamily, appears to be uniquely adapted for the metabolism of DMSP. DmdD has a crystal structure largely similar to that of other crotonases, a hexamer made up of a dimer of trimers. **Figure 5** shows an overlay of one DmdD monomer with a monomer of rat liver enoyl-CoA hydratase (ECH). DmdD is similar to the rat liver ECH, sharing 32% amino acid identity. The main difference is that in DmdD the C-terminal loops of one of the trimers is oriented so that it can interact with the phosphate groups of CoA (**Figure 5**) (Tan et al., 2013). The same glutamate residues that are conserved and important for catalysis in the rat liver ECHs are also conserved in DmdD. However, DmdD is not nearly as efficient as an ECH at catalyzing the hydration of crotonyl-CoA, with a catalytic efficiency of 2100 mM^-1^s^-1^ compared with the typical values of 45000–119000 mM^-1^s^-1^ of other crotonases. DmdD instead displays a *K*_m_ of 0.008 mM for MTA-CoA and a high catalytic efficiency, 5400 mM^-1^s^-1^ (Kiema et al., 1999; Feng et al., 2002; Tan et al., 2013). This greater catalytic efficiency only applies to reactions with MTA-CoA and appears to be due, at least in part, to the structure of MTA-CoA. The combination of the double bond and sulfur atom in MTA-CoA appear to be key for high rates DmdD hydrolysis activity as reactions with MMPA-CoA and crotonyl-CoA occur at lower rates (Tan et al., 2013).

While DmdD is highly efficient at catalyzing the hydration of MTA-CoA, it is not widely distributed (Reisch et al., 2011b). DmdD is absent from the majority of marine bacteria that utilize the demethylation pathway, i.e., possess DmdA (**Figure 3**). An ortholog of DmdD has been identified in the DmdD negative *R. lacuscaerulensis* as well as in *R. pomeroyi* (Reisch et al., 2011b). This enzyme, now designated AcuH for acrylate utilization hydratase, is an ECH with high activity toward acryloyl-CoA and crotonyl-CoA, but also displays activity toward MTA-CoA. The designation is similar to that of the acryloyl-CoA reductase AcuI. As a result of its activity toward acryloyl-CoA and MTA-CoA, AcuH is predicted to play an important role in the metabolism of acrylate formed from the cleavage pathway as well as MTA-CoA formed from the demethylation pathway (**Figure 2**) (Sullivan et al., 2011; Todd et al., 2012a; Reisch et al., 2013). By contrast, DmdD has no activity toward acryloyl-CoA. AcuH is less efficient than DmdD at hydrolyzing MTA-CoA, however, it is far more common, being found in a wide range of microorganisms, including those in the Roseobacter clade (**Figure 3**). AcuH appears to be a more versatile enzyme than DmdD and has maintained more of its functional similarity to other ECHs. Since AcuH likely functions in both the cleavage and the demethylation pathways, this strategy gives the cells increased metabolic flexibility and may also protect against acryloyl toxicity. DmdD, by contrast, has adapted specifically to function in the demethylation pathway, possibly allowing organisms which possess DmdD to utilize DMSP more efficiently.

## Links Between the Bacterial DMSP Cleavage and Demethylation Pathways

Interactions between the cleavage pathway and demethylation pathways in organisms that contain both are an ongoing field of study. One proposal is that a ‘bacterial switch’ allows bacteria possessing both pathways to alternate between producing more or less DMS and MeSH (Kiene et al., 2000; Simo, 2001). While there is currently no consensus as to what signal controls the switch, the identification of the acrylate utilization enzymes AcuH and AcuI has begun to shed light on the topic. AcuH, as mentioned above, may function in both the cleavage and demethylation pathways (Reisch et al., 2011b, 2013). AcuI is an acryloyl-CoA reductase whose gene has been found immediately downstream of *dmdA* in many members of the Roseobacter clade. In *R. pomeroyi, dmdA* and *acuI* are co-regulated with acrylate acting as an inducer (Sullivan et al., 2011; Todd et al., 2012a). Since acrylate and acryloyl-CoA are inhibitory for bacterial growth, it has been proposed that AcuI maintains cellular acrylate concentrations below inhibitory levels. Thus, when acrylate concentrations increase as a result of DMSP lyase activity, AcuI and DmdA co-regulation results in increased activity of both enzymes. Elevated AcuI activity then alleviates inhibition caused by the build-up of acrylate, while increases in DmdA activity stimulate the demethylation pathway, allowing DMSP to be utilized in a manner that does not produce acrylate (Todd et al., 2012a). The activity of the demethylation pathway may also respond to carbon and energy limitation, with regulation resulting from changes in the energy charge of the cell (Bullock et al., 2014). Further research is still needed to investigate the physiological cues for balancing the demethylation and cleavage pathways.

## Conclusion: Evolution of DMSP Metabolism

It is unclear what the original impetus for the development of DMSP biosynthetic pathway may have been. The proposed roles for DMSP in marine phytoplankton as an osmolyte, antioxidant, predator deterrent, cryoprotectant, and as an energy overflow mechanism each could provide great benefits, particularly in consistently changing marine environments. If DMSP was originally produced as part of an overflow mechanism for dealing with unbalanced growth due to nutrient limitation, the other benefits provided by this compound may have selected for the maintenance of this pathway. The case has been made for the co-evolution of the marine Roseobacter and the DMSP producing-phytoplankton. Members of the Roseobacter clade are abundant in coastal waters and are one of the main bacterial groups enriched during DMSP-producing phytoplankton blooms (Gonzalez et al., 2000; Zubkov et al., 2001; Moran et al., 2007). Based on independent time estimates assisted by the cyanobacterial fossil calibration and estimates derived from the mutation rate clock method, the Roseobacter ancestor likely underwent a genome expansion, coincident with the increase in abundance and diversification of the dinoflagellates and coccolithophores around 250 mya (Luo et al., 2013; Luo and Moran, 2014; Sun et al., 2017). Thus, the radiation of dinoflagellates and coccolithophores may have provided new environments for members of the Roseobacter clade, in much the same way that the breakup of Pangea and changes in ocean redox chemistry created new environments for the proliferation of the red plastid lineage members (Whitfield, 2001; Quigg et al., 2003; Luo et al., 2013).

Research into the diversity of bacterial DMSP utilization enzymes and their regulation is still ongoing. New Roseobacter isolates showing adaptations to their particular environmental niches are continually being discovered. Recently, two new members of the Roseobacter clade were isolated from deep-sea water, *Thiobacimonas profunda* JLT2016 and *Pelagibaca abyssi* JLT2014. While these isolates did not possess DMSP metabolic genes, their genomes included genes for inorganic sulfur oxidation and CO_2_ fixation, further demonstrating the metabolic flexibility of this clade (Tang et al., 2016) and illustrating that members of the Roseobacter clade have exploited different routes to metabolically thrive in their environments (Luo et al., 2014). Since the first Roseobacter genome expansion 250 mya, many factors could have played a role in the development of metabolic pathways for the utilization of a specific carbon source like DMSP. There was not one path for these organisms to follow but many, allowing for a diversity of solutions to a single goal. As DMSP became more readily available in the environment, marine organisms, such as *R. pomeroyi* and other Roseobacters, likely adapted to utilize this compound as a source of carbon as well as reduced sulfur. Members of the Roseobacter clade were well poised for this task, being metabolically versatile bacteria and thus able to thrive in dynamic environments (Moran et al., 2007, 2012). Exposure to DMSP would be the driving force behind this evolution of function, putting pressure on organisms to adapt proteins already encoded in their genomes to utilize this new compound. Possible examples of this can be seen in the enzymes DmdA and DmdD from the demethylation pathway and DddP from the cleavage pathway. Each of these enzymes became more specialized to function in DMSP metabolism. Although structurally similar to their likely ancestral enzymes, they have undergone major changes in substrate specificities and, in some cases, enzymatic mechanism. AcuH seems to have adopted a different strategy and can function as both a MTA-CoA hydratase in the demethylation pathway and an acryloyl-CoA hydratase in acrylate metabolism. DmdB and DmdC also maintained activity with a wide range of substrates while adapting to function efficiently in DMSP metabolism as well. In these latter cases, there appears to have been minimal adaptations to DMSP metabolism, with changes in regulatory strategy as well as small changes in substrate specificity to accommodate a novel substrate.

Enzymes are known to diverge from a parental function to develop new substrate specificities, often via the duplication of genes encoding a multifunctional and multispecific enzymes that then undergo an alteration in substrate specificity (Noda-Garcıa et al., 2013). Although a certain core set of amino acid residues are required for functionality and structure, there is also room for variation and change, allowing evolution of new functions and substrate specificities (Perona and Craik, 1997). Thus, the amino acid sequences and structures of the enzymes catalyzing the DMSP catabolic reactions do not vary greatly from their non-DMSP degrading counterparts. From this perspective, the enzymes of the DMSP demethylation and cleavage pathways are examples of the various processes of enzyme adaptation and evolution that occurred within the Roseobacter clade in the last 250 million years.

## Author Contributions

WW and HB were responsible for the conceptualization and design of this manuscript. HB and HL collected data and analyzed the literature for this review. HB drafted the original manuscript. WW and HL reviewed and edited the manuscript. HB, WW, and HL provided final approval of the manuscript prior to submission.

## Conflict of Interest Statement

The authors declare that the research was conducted in the absence of any commercial or financial relationships that could be construed as a potential conflict of interest.
